# Many steps can be taken to enhance recovery after thoracic surgery

**DOI:** 10.1186/s13019-021-01655-z

**Published:** 2021-09-28

**Authors:** Eliza Sophie Hartmann, Paul Philipp Heinisch, Markus M. Luedi, Maks Mihalj

**Affiliations:** 1 Department of Anesthesiology, Langenthal Regional Hospital, Bern, Switzerland; 2 Department of Cardiovascular Surgery, Bern University Hospital, University of Bern, Bern, Switzerland; 3 Department of Anaesthesiology and Pain Medicine, Bern University Hospital, University of Bern, Bern, Switzerland; 4 Department of Congenital and Pediatric Heart Surgery, German Heart Center Munich, Technical University Munich, Munich, Germany

**Keywords:** Enhanced recovery after thoracic surgery, ERAS, Cardiothoracic surgery, Operating room management, Optimization, Staffing, Shared decision making, Non-technical skills

## Abstract

Our letter to the editor comments on issues raised in the May 14, 2020, article by Budacan et al. addressing the development of enhanced recovery after thoracic surgery. In the United Kingdom and Ireland, a nationwide survey identified issues. Here, we expand on the authors’ findings.

## To the Editor

We read with great interest the recent article by Budacan et al. describing the variations and difficulties in implementing the current guidelines for enhanced recovery after thoracic surgery (ERAS) in the United Kingdom and Ireland. Through a nationwide survey and using simple descriptive statistics, the authors captured the unfiltered reality of variations in the practice of the ERAS guidelines by a group of medical professionals [1]. The main variations and differences were observed at staffing levels, and included inconsistent teamwork, limited availability of resources over the weekend, and reduced access to established smoking cessation programs, to name a few. While the authors suggested that educating the staff and improving communication and teamwork could improve ERAS in thoracic surgery, we believe that an interdisciplinary approach should also involve operating room (OR) management, human resources, and staffing, as already in practice by some [2].

Good OR management might also include matched allocation of anesthesiologists, not only to an individual patient but also to a specific surgeon, as this has been shown to improve team performance, and therefore has a positive effect on operating room efficiency through better turnaround times [3]. Further points to consider are the perioperative team setup, patient age, and American Society of Anesthesiology (ASA) risk assessment, as these have been shown to influence OR times [4]. Yang et al. demonstrated that forming dedicated ERAS groups for cardiac surgery increases recovery after cardiac surgery, and should include not only the surgeon, but also the anesthesiologist, the nurses, and the administration [5]. This ultimately helps build team consensus, and supports the consistent use of ERAS by all team members.

Given the comorbidity and risk profiles of cardiothoracic surgery patients, preoperative assessment and detailed planning should include the surgeon as well as the anesthesiologist and other applicable specialists. Preoperative optimization of the patient should be performed whenever possible, including management of medication, coagulation and blood status, anticipated blood management, as well as standardized programs for enhancing the physical and nutritional status [6]. Shared decision-making should involve the surgeon, the anesthesiologist and the patient, and might further improve the implementation of ERAS.

Last but not least, Loup et al. demonstrated the importance of non-technical skills (NTS) in the multidisciplinary care of surgical patients. These involve personal and social skills, as well as the cognitive abilities of all team members, including the patient [7]. It is well known that technical skills alone are not sufficient to produce good outcomes in surgical fields, and intraoperative complications or errors may occur not only due to insufficient technical skills, but also due sub-standard commitment and organizational influences, to name only a few [8]. Training programs aimed at increasing the NTS should be offered by all hospital systems. These should address leadership, communication, teamwork and resource management. As these were the main factors identified in the study by Budacan et al., their implementation might further increase the success and consistency of ERAS in (cardio-) thoracic surgery.

## Data Availability

Not applicable.

## Abbreviations

ASA: American Society of Anesthesiology
ERAS: Enhanced Recovery After Thoracic Surgery
NTS: Non-technical skills
OR: Operating room
